# Classification of journals in the QUALIS System of CAPES – URGENT need of changing the criteria!

**DOI:** 10.1590/S1808-86942010000200001

**Published:** 2015-10-19

Due to its concern about the future of Brazilian scientific journals after new criteria were adopted by the QUALIS system of CAPES (Brazilian Federal Agency for the Improvement of Higher Education), the Brazilian Medical Association (Associação Médica Brasileira - AMB) has held several meetings at its headquarters in São Paulo to discuss this matter. Editors of the main Brazilian medical journals, directors of the Brazilian Association of Scientific Editors (Associação Brasileira de Editores Científicos – ABEC), and coordinators of the areas Medicine II and Medicine III of CAPES exchanged information and came out with proposals aimed at improving the evaluation process of Brazilian scientific journals by the new QUALIS system of CAPES. The classification of the scientific production according to the QUALIS system will be one of the main items of the three-year evaluation of graduate programs. Since most scientific articles published in Brazilian journals are produced within graduate programs supported by CAPES, it was very important to fine tune the speech and make sure that all the parties involved speak the same language. The editors of scientific journals are afraid that the new criteria adopted by CAPES may create a subgroup of journals exclusively based on the ISI Impact Factor. The previous criterion recommended an impact factor of 1 as a cutoff point. Recently, some Brazilian journals have achieved this goal after putting a great load of effort into it. However, in addition to considering only the impact factor, the new criteria established much higher cutoff points. If this measure is adopted, Brazilian journals will be despised by graduate academic advisors and students – who are the main producers of science in Brazil – thus creating a vicious cycle within which Brazilian journals will hardly survive.

Professor João Pereira Leite spoke on behalf of CAPES. In addition to being the coordinator of the area Medicine II, he is also the current representative of the health area in the Technical Scientific Council, which is the main department of CAPES. During one of the meetings, professor Leite provided a detailed explanation about the criteria adopted for three-year evaluation and their impact on Brazilian graduate programs. He also explained that, in face of the evident improvement of the quality of graduate programs, it was necessary to increase the cutoff point or the separation point in order to better differentiate these programs and classify them in terms of their quality level. Based on data from the graduate programs – collected using the data collection system of CAPES – it was found that many programs had more than 50% (some of them even had 80%) of their scientific production published in journals classified at higher levels of the classification scale. On its turn, CAPES decided to create a larger number of levels with the purpose of reclassifying the Brazilian journals. A decreasing scale based on the impact factor has been suggested: A1, A2, B1, B2, B3, B4, B5, and C. In addition, CAPES also created an equivalence factor according to which the number of articles published in journals belonging to the lower levels of the scale would be equivalent to a smaller number of articles published in journals belonging to the higher levels of the classification scale. Therefore, for example, for a certain area, 2 articles B1 would be equivalent to 1.2 article A1; 1 article B1 + 1 article A2 would be equivalent to 1.4 article A1; 3 articles B2 would be equivalent to 1.2 article A1. According to professor Leite: “Such equivalence would bring benefits for journals with different qualification levels.” Professor Leite also informed that the new classification system was designed based on the median of the journals' impact factor provided by the Journal Citation Reports (JCR) and calculated every year by the ISI Web of Knowledge. A list of journals including each area of CAPES was made to calculate the median. The median for each area was based on this list and on the respective impact factors; then, a new classification system ranging from A1 to C was created.

The editors reminded professor Leite that the three-year evaluation process of CAPES would cause a relative disagreement for the reclassification of the journals, since several Brazilian journals will have their impact factor increased or published for the first time during 2010, mainly those that have just been indexed in the ISI. In addition, these journals would have to wait for three years to change their classification in the new QUALIS! Another aspect that was questioned by the editors is related to the choice of the impact factor published by the JCR as the ONLY and universal index to assess the quality of the journals. There is a high standard deviation in the impact factors of different journals. Certainly, that is the reason why CAPES used the median of these indexes to analyze the scientific production of graduate programs. Actually, according to this criterion, some medical specialties, such as those related to surgery, have their best journals with a lower impact factor, which might result in a bias that could be extremely unfavorable for them.

Both the editors and CAPES agree that valuing the Brazilian journals is important for the Brazilian scientific growth and development. With the purpose of keeping and stimulating this virtuous cycle, it is necessary to promote and foster the citation of articles published by Brazilian authors, intensify the efforts of editors, reviewers and authors to increase the quality of the articles and, on the other hand, make sure that the governmental agencies, especially CAPES and CNPq, provide support for the management of the financial resources and qualitative classification of the journals.

The results of these discussions were presented in several meetings attended by editors, coordinators of graduate programs and researchers, and new suggestions were made. The ideas described below will be used as the conclusion of this editorial and, at the same time, we hope that they serve as an important tool to convince the agencies to change the criteria of journal classification in the QUALIS system of CAPES. Our suggestions are as follows:

• The qualitative analysis of the Brazilian journals should be reassessed and it should not include only the Impact Factor published by the JCR;
• The specific characteristics of each area of interest or each specialty should be taken into consideration and respected;
• The Brazilian publishing industry, in contrast with what happens in other countries where it is financed by private investors, is financially supported by public and private universities and scientific associations;
• Brazilian journals need to receive more support and stimuli, which may be provided as: financial remuneration for editors, financial support for journals, greater visibility for national journals abroad, more objective and encompassing criteria for the qualitative classification, and support based on the performance of each journal;
• Support for the internationalization of scientific journals by fostering the professionalization of the editorial process and promotion of the journals in other countries;
• Continuous update of the journal classification system within the new QUALIS with no need to wait for the three-year period of assessment;
• Participation of scientific associations (ABEC, AMB, among others) in the decision-making process regarding the QUALIS system of CAPES;
• Strong stimulation of citations directly in the source of scientific production, that is, graduate programs (for instance, recommending that graduate programs classified as 6 or 7, in addition to being required to have a percentage of articles published in journals with high impact factor, should also have a percentage of articles published in Brazilian journals. This measure includes both ends of the scientific production, since young and future researchers begin their careers publishing in national journals under the supervision of experienced researchers.

In conclusion, to show its agreement with all these measures and its concern with the consequences of the new QUALIS of CAPES and other evaluation procedures of journals, ABEC devoted three days to the forum of the areas during its last National Meeting of Scientific Editors, which was held in November 2009. During this meeting, members of the staff of CAPES and editors of all the areas of scientific knowledge held long discussions on this topic and came up with the suggestion of the Forum of the Areas Guidelines of the 12th National Meeting of Scientific Editors – 2009, which will be timely sent to all the Brazilian sponsoring agencies, which should be done periodically because this is a continuous process.

The following editors approved this editorial:

**Table:** 

Adagmar Andriolo	Jornal Brasileiro de Patologia e Medicina Laboratorial
Aécio Flávio Meireles Souza	Revista GED
Alberto Queiroz Farias	Revista Arquivos de Gastroenterologia
Alfredo José Afonso Barbosa	Jornal Brasileiro de Patologia e Medicina Laboratorial
Antonio Spina França Netto	Revista ARQUIVOS DE NEURO-PSIQUIATRIA
Arnaldo José Hernandez	Revista Brasileira de Medicina do Esporte
Aroldo F. Camargos	Revista Femina
Benedito Barraviera	Journal of Venomous Animals and Toxins including Tropical Diseases
Bogdana Victoria Kadunc	Surgical & Cosmetic Dermatology da Soc. Brasileira de Dermatologia
Bruno Caramelli	Revista da Associação Médica Brasileira
Carlos Eduardo Aguilera Campos	Revista Brasileira de Medicina de Família e Comunidade
Carlos Brites	Brazilian Journal of Infectious Diseases
Dejair Caitano do Nascimento	Hansenologia Internationalis
Domingo M. Braile	Revista Brasileira de Cirurgia Cardiovascular
Dov Charles Goldenberg	Revista Brasileira de Cirurgia Plástica
Edmund Chada Baracat	Revista da Associação Médica Brasileira
Edna T Kimura	Arquivos Brasileiros de Endocrinologia e Metabologia
Edson Marchiori	Revista Radiologia Brasileira
Eduardo de Paula Vieira	Revista Brasileira de Coloproctologia
Eros Antônio de Almeida	Revista da Sociedade Brasileira de Clínica Médica
Geraldo Pereira Jotz	Revista Brasileira de Cirurgia Cabeça e Pescoço
Gilberto Camanho	Revista Brasileira de Ortopedia
Gilberto Friedman	Revista Brasileira de Terapia Intensiva
Giovanni Guido Cerri	Radiologia Brasileira
Ivomar Gomes Duarte	Revista de Administração em Saúde
Izelda Maria Carvalho Costa	Anais Brasileiros de Dermatologia
João Ferreira de Mello Júnior	Brazilian Journal of Otorhinolaryngology
Joel Faintuch	Revista Brasileira de Nutrição Clínica
José Antônio Baddini Martinez	Jornal Brasileiro de Pneumologia
José Antonio Livramento	Revista Arquivos de Neuropsiquiatria
José Eduardo Ferreira Manso	Revista do Colégio Brasileiro de Cirurgiões
José Luiz Gomes do Amaral	Revista da Associação Médica Brasileira
Linamara Rizzo Battistella	Revista Acta Fisiátrica
Luís dos Ramos Machado	Revista Arquivos de Neuropsiquiatria
Luiz Felipe P. Moreira	Arquivos Brasileiros de Cardiologia
Luiz Henrique Gebrim	Revista Brasileira de Mastologia
Marcelo Madeira	Revista Brasileira de Mastologia
Marcelo Riberto	Revista Acta Fisiátrica
Marcus Bastos	Jornal Brasileiro de Nefrologia
Mário Cícero Falcão	Revista Brasileira de Nutrição Clínica
Mario J. da Conceição	Revista da Sociedade Brasileira de Anestesiologia
Mauricio Rocha e Silva	Revista Clinics
Milton Artur Ruiz	Revista Brasileira de Hematologia e Hemoterapia
Milton K. Shibata	Arquivos Brasileiros de Neurocirurgia
Mittermayer Barreto Santiago	Revista Brasileira de Reumatologia
Nelson Adami Andreollo	Arquivos Brasileiros de Cirurgia Digestiva
Osvaldo Malafaia	Arquivos Brasileiros de Cirurgia Digestiva
Regina Helena Garcia Martins	Brazilian Journal of Otorhinolaryngology
Renato Soibelmann Procianoy	Jornal de Pediatria
Ricardo Baroudi	Revista Brasileira de Cirurgia Plástica
Ricardo Fuller	Revista Brasileira de Reumatologia
Ricardo Guilherme Viebig	Arquivos de Gastroenterologia
Ricardo Nitrini	Dementia & Neuropsychologia
Rita Cristina Mainieri R. de Moura	Revista da Associação Brasileira de Medicina de Tráfego
Rogério Dedivitis	Revista Brasileira de Cirurgia Cabeça e Pescoço
Ronaldo Damião	Urologia Contemporânea
Sergio Lianza	Revista Medicina de Reabilitação
Sigmar de Mello Rode	Brazilian Oral Research
Winston Bonetti Yoshida	Jornal Vascular Brasileiro
Zuher Handar	Revista Brasileira de Medicina do Trabalho

